# Fabrication of Eco-Friendly Solid-State Symmetric Ultracapacitor Device Based on Co-Doped PANI/GO Composite

**DOI:** 10.3390/polym11081315

**Published:** 2019-08-06

**Authors:** Hajera Gul, Anwar-ul-Haq Ali Shah, Salma Bilal

**Affiliations:** 1 Conductive Polymers and Electrochemistry Laboratory, National Centre of Excellence in Physical Chemistry, University of Peshawar, Peshawar 25120, Pakistan; 2 Physical Chemistry Laboratory, Institute of Chemical Sciences, University of Peshawar, Peshawar 25120, Pakistan; 3 TU Braunschweig Institute of Energy and Process Systems Engineering, Franz-Liszt-Straße 35, 38106 Braunschweig, Germany

**Keywords:** polyaniline, graphene oxide, composite, co-doped, solid-state symmetric ultracapacitor, specific capacitance

## Abstract

An eco-friendly solid-state symmetric ultracapacitor (Uc) device was fabricated using a polyaniline graphene oxide composite co-doped with sulfuric acid (H_2_SO_4_) and dodecyl benzene sulfonic acid (DBSA) or camphor sulfonic acid (CSA), as electrode material utilizing gold sheets as current collectors. The device showed specific capacitance value of 150 F/g at 1 A/g current density, with a capacitance retention value of 93.33% at higher current density (10 A/g), indicating a high rate capability. An energy density of 15.30 Whkg^−1^ with a power density of 1716 Wkg^−1^ was obtained at the current density of 1 A/g. The values of areal capacitance, power density, and energy density, achieved at the current density of 5 mAcm^−2^, were 97.38 mFcm^−2^, 9.93 mWhcm^−2^, and 1.1 Wcm^−2^, respectively. Additionally, the device showed very low solution and charge transfer resistance (0.885 Ω and 0.475 Ω, respectively). A device was also fabricated utilizing copper as current collector; however, a lower value of specific capacitance (82 F/g) was observed in this case.

## 1. Introduction

To utilized, interrupted/or intermittent power outputs from natural renewable energy resources, it is necessary to evolve efficient energy storage systems [1]. Batteries and supercapacitors (SCs), also known as ultracapacitors (Ucs), are most efficient among the various energy storage devices. Ultracapacitors are superior over batteries regarding their high-power density. High-stability, fast charge-discharge cycles, high coulombic efficiency, and environment friendly nature are the prime advantages of Ucs over lithium ion batteries, which make them ideal energy storage devices. However, when liquid electrolytes are used in conventional Ucs devices, it raises safety concerns and demands for high-standard safety encapsulation materials and technologies. Moreover, the liquid electrolytes release some hazardous byproducts into the environment [2]. This problem can be solved by replacing liquid electrolyte in these devices with a solid counterpart. Solid electrolytes can open the way for lightweight, thin, economically viable, and flexible future devices.

Solid-state Ucs are composed of electrodes, solid electrolyte, and separator to a solid whole. These Ucs are preferred over their liquid counterparts due to their light-weight, high-safety, high-flexibility, and eco-friendly nature [3,4]. Solid-state Ucs devices overcome the possibility of leakage of hazardous byproduct into the environment which is one of the major drawbacks associated with liquid electrolytes.

Electrode materials for Ucs are generally divided into two categories [5,6]. One is carbon-based materials where the charge is stored in the electrical double layer. Examples of such materials are graphene oxide (GO) or graphene, porous carbon, carbon foam, nanotubes or fiber, activated carbon, etc. [7]. Their capacitance is mostly dependent on the specific surface area. These materials often show a stable long-term cycling performance. However, they have low capacitance value. The Second category of electrode materials include pseudocapacitive materials where the charge storage arises from redox reactions. These materials generally include conductive polymers, metal oxides, hydroxides, nitrides, and sulfides. Polyaniline (PANI), a member of the class of conductive polymers, possess high pseudocapacitance with the advantages of low cost, easy synthesis, and high conductivity [8,9]. However, PANI has the limitation of poor cycling stability. A combination of the two categories of electrode materials in the form of composites can offer helpful solutions to overcome the problems associated with both materials. Their composite can integrate their best properties. Nanocomposites of PANI with GO have been reported to show enhanced physical and chemical properties compared with pristine PANI or GO and can be used in various applications [10]

Mostly gel electrolytes are used in solid state Ucs [1]. However, gel electrolytes have limitations, such as low electrode-electrolyte interfacial area and poor charge storage properties. Due to the decline in surface area, the charge storage properties decrease because high contact resistance arises from the low integrity of the electrode-electrolyte material. For storage device, very low equivalent series resistance is a crucial factor. Nowadays, researchers are working to get free-standing electrode materials to fabricate flexible solid-state devices. To replace liquid electrolytes in Ucs, such an electrode-electrolyte interface is needed that mimics the liquid-solid interface in the conventional systems.

Polyvinyl alcohol (PVA)–H_2_SO_4_ gel, which is known for its high conductivity and flexibility, was employed as polymer electrolyte [1]. If the electrode material is porous, PVA–H_2_SO_4_ can be successfully intercalated. Also, the presence of water plays an important role in reducing the density of PVA which further facilitate its easy penetration into the micropores of the electrode material. The surface layer of this electrolyte can function as a separator between the electrodes when the two electrodes are combined to form a single cell unit.

In the present study, we tried to overcome various limitations of solid-state Ucs, by utilizing two different composites of conductive polyaniline with graphene oxide, where the composite was co-doped with sulfuric acid and dodecylbenzene sulfonic acid (DBSA), or camphor sulfonic acid (CSA). Pure PANI is an insulator but addition of dopants, CSA, DBSA, and H_2_SO_4_ can enhance the electrical conductivity by many orders. To overcome leakage problems, (PVA)–H_2_SO_4_ gel was used as electrolyte to assemble solid-state symmetric ultracapacitor devices. It is a water soluble and biodegradable polymer and minimization of electrolyte leakage from the device can be considered an effort to make the device eco-friendlier. Furthermore, efforts were made to reduce the overall cost of the energy storage devices. So, the composite with best properties was tested for its capacitive properties with copper as current collector to assemble solid state symmetric device.

## 2. Experimental

### 2.1. Materials

Aniline (C_6_H_5_NH_2_), Sigma-Aldrich (Hamburg, Germany), was double distilled under reduced pressure and stored in a refrigerator. Chloroform (CHCl_3_), sulfuric acid (H_2_SO_4_), and hydrochloric acid (HCl) were purchased from Scharlau Chemie S.A (08181 Sentmenat, Spain) and used as received. Dodecyl benzene sulfonic acid (C_12_H_25_C_6_H_4_SO_3_H), ammonium persulfate ((NH_4_)_2_S_2_O_8_)), graphite powder, potassium permanganate (KMnO_4_), sodium nitrate (NaNO_3_), hydrogen peroxide (H_2_O_2_), *N*,*N*–Dimethylformamide (DMF), camphor sulfonic acid (CSA), polytetrafluoroethylene (PTFE), and acetone were purchased from Sigma–Aldrich (Hamburg, Germany). All these chemicals were research grade and used without further purification.

### 2.2. Synthesis of Co-Doped Polyaniline Graphene Oxide Composites

Graphene oxide (GO) was synthesized by modified Hummer’s method [11] and 0.090 g of it was dispersed in distilled water (20 mL) through sonication. Sonication was carried out for 30 min to form homogeneous dispersion [12]. In a round-bottom flask, 2.3 mL of dodecylbenzenesulfonic acid (DBSA) was added to 50 mL of chloroform under constant stirring. Then, 1.5 mL of aniline was added to the above reaction mixture, followed by dropwise addition of 25 mL of 1.1 M H_2_SO_4_ and 25 mL of 0.09 M ammonium persulfate (APS). Graphene oxide dispersion was then added slowly to the above reaction mixture and reaction was allowed for 24 h under constant stirring. Green precipitate of co-doped polyaniline graphene oxide (ds@PANI/GO) composite was obtained. It was washed with distilled water 3 times and then with acetone to remove unreacted species. Green precipitate was filtered and dried in an oven at 60 °C, while co-doped cs@PANI/GO composite was obtained via the same procedure using camphor sulfonic acid (CSA) instead of DBSA.

### 2.3. Fabrication of Symmetric Solid-State Supercapacitor Device

To fabricate the solid-state Uc device, gel electrolyte ((PVA)–H_2_SO_4_ gel), was used. This gel electrolyte was prepared by adding PVA powder slowly into 60 mL aqueous solution of 1 M H_2_SO_4_ under vigorous stirring. The clear solution was then kept for 1 h at 85 °C without stirring. After that, two identical electrodes coated with composite materials were immersed into the PVA–H_2_SO_4_ electrolyte for 5 min to allow good accessibility of electrolyte ions into the active materials. The Uc was assembled by sandwiching them with a filter paper as separator.

### 2.4. Characterization

The synthesized samples were characterized through the following techniques:

Ultraviolet visible (UV–Vis) spectroscopic analysis was carried out in chloroform using Perkin Elmer spectrophotometer (Buckinghamshire, UK) having cell of quartz of 1 cm path length. To check the thermal degradation pattern, structural aspects, and thermal stability of composites, thermogravimetric analysis (TGA) was carried out. It was executed by making use of Perkin Elmer (Waltham, MA, USA) at a heating rate of 10°/min under N_2_ atmosphere.Fourier transform infrared (FTIR) spectra were recorded with IR Affinity-S1 spectrophotometer (Shimadzu, Tokyo, Japan), scanning over the wavenumber range of 400–4000 cm^–1^, with 2 cm^–1^ resolution. X-ray diffraction (XRD) patterns of samples were recorded utilizing Cu Kα radiations (λ = 1.5405 A°) on a Rigaku (JEOL, Tokyo, Japan) X-ray diffractometer with a progressive scan rate of 0.05°/s. Surface imaging and elemental mapping of the synthesized samples were performed through scanning electron microscopy (SEM) and SEM–Energy Dispersive X-ray (SEM-EDX) analysis (Helios G4 CX DualBeam microscope equipped with Octane Elite, EFI Berlin Germany). The size of the particles was determined using Nano Measurer 1.2.5 software.

Electrochemical characterization of composites was conducted in an electrochemical cell utilizing three electrodes using 3000 ZRA potentiostat/galvanostat Gamry (Warminster, PA, USA). Eighty percent PANI–GO composite, 10% activated carbon, and 10% PTFE were dispersed in DMF and coated on a gold working electrode. Whereas, a coiled wire of gold and saturated calomel electrode (SCE) were utilized as a counter electrode and as a reference electrode, respectively; 1 M H_2_SO_4_ was utilized as an electrolyte. In a 3 electrode setups, CVs were accomplished in potential limits from −0.2 to 0.8 V at 10 mV/s. For solid-state symmetric device CV and galvanostatic charge discharge (GCD), analysis was carried out in the potential range of 0 to 0.9 V at scan rates of 10–500 mV/s and current densities of 1–5 A/g, respectively. Total mass of composites deposited on both the gold sheet electrodes was 5.2 mg. Equations (1)–(5) were respectively used for calculation of capacitance (C), specific capacitance (*C_s_*), energy density, power density, and areal capacitance (*C_A_*). Where *I* was applied current, Δ*V* was potential window used, total mass of both electrodes (*M*), and total area of both electrodes (*A*).

(1) $$C=\frac{I\;\times \;\Delta t}{\Delta V}$$

(2) $$C_{s}=\frac{4\;\times \;C}{M}$$

(3) $$E=\frac{1}{2\times 3.6}C\Delta V^{2}$$

(4) $$P=\frac{E\times 3600}{\Delta t}$$

(5) $$C_{A}=\frac{I\times \Delta t}{A\times \Delta V}$$

## 3. Results and Discussion

### 3.1. UV-Visible Spectroscopic Analysis

**Figure 1:**
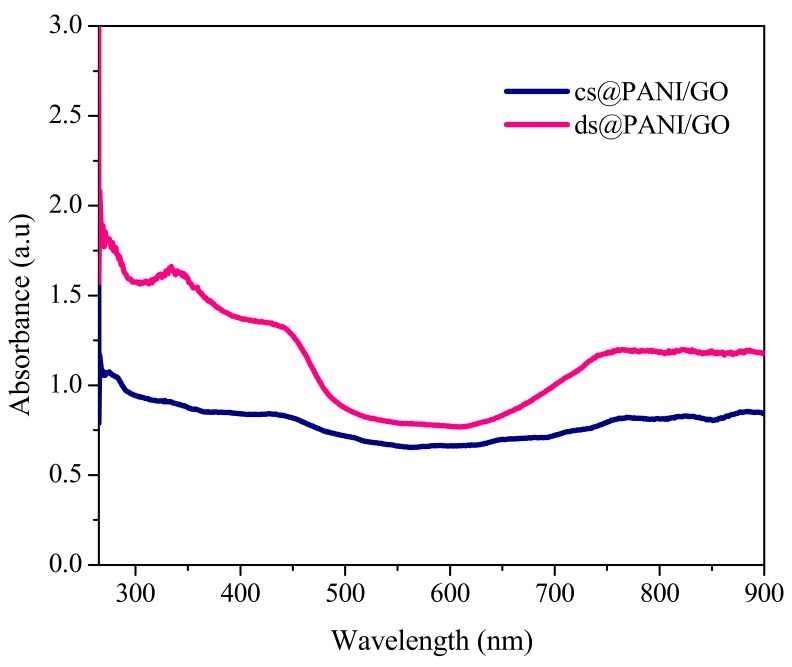
UV-Visible spectra of dodecylbenzenesulfonic acid-sulfuric acid-polyaniline graphene oxide (ds@PANI/GO) and camphor sulfonic acid-sulfuric acid-polyaniline graphene oxide (cs@PANI/GO).

Figure 1, depicts the UV-Vis spectra of co-doped cs@PANI/GO and ds@PANI/GO composite in chloroform. The peaks present at 279 (co-doped cs@PANI/GO), 274 (co-doped ds@PANI/GO), and 776 nm are typical peaks of PANI in its doped form and are ascribed to polaron-π* and π-polaron transitions, respectively [13]. The absorption peak of π-polaron transition is broader and also emerges at high wavelength indicating a high doping level of the material.

The bands at 331 (co-doped ds@PANI/GO) and 336 nm (co-doped cs@PANI/GO) were due to the restricted polaron band for protonated structure of polymer in these composite materials [14].

### 3.2. FTIR Characterization

**Figure 2:**
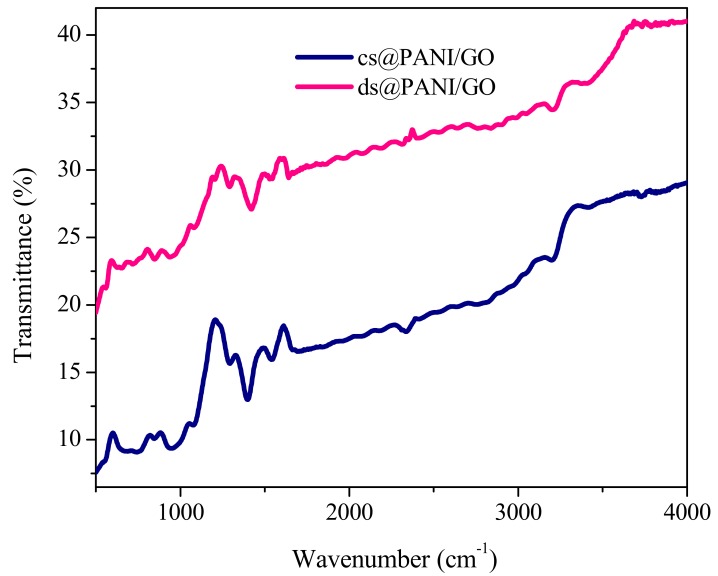
FTIR spectra of ds@PANI/GO and cs@PANI/GO, as indicated.

The FTIR spectra of ds@PANI/GO and cs@PANI/GO composites are given in Figure 2. Both the composites show peaks that are characteristic of PANI. Bands appearing at 1427 cm^−1^, 1546 cm^−1^, and 1214 cm^−1^ for ds@PANI/GO were due to the C–C stretching of the quinoid/and benzenoid moieties and C=N stretching vibrations of PANI [15]. The spectrum also affirmed strong absorption bands at 1546 cm^−1^ and 1427 cm^−1^ that were the consequence of the stretching mode of vibrations of C=C in the quinonoid and benzenoid rings, respectively. The band appearing at 1200 cm^−1^ were the evidence of the presence of aromatic C–N, C=N, and C–H stretching modes of vibrations in the polaron framework of PANI. The band at 670 was due to the symmetric and asymmetric stretching vibrations of the O=S=O and S–O groups due to the DBSA or CSA. The band at 1002 cm^−1^ indicates NH^+^…SO^–^_3_ interaction between PANI and the dopants [16]. In these composites, the appearance of the peaks at 2320 cm^–1^ and 1078 cm^−1^/3028–3500 cm^−1^ due to the O=C–O and OH groups, respectively, manifested the existence GO in the composite. The peak at about 3409 cm^−1^ in these composites can be assigned to overlap of O–H stretching vibration of GO and N–H stretching of PANI [17] indicating the existence of a conductive composite framework [15].

### 3.3. X-ray Diffraction Analysis

**Figure 3:**
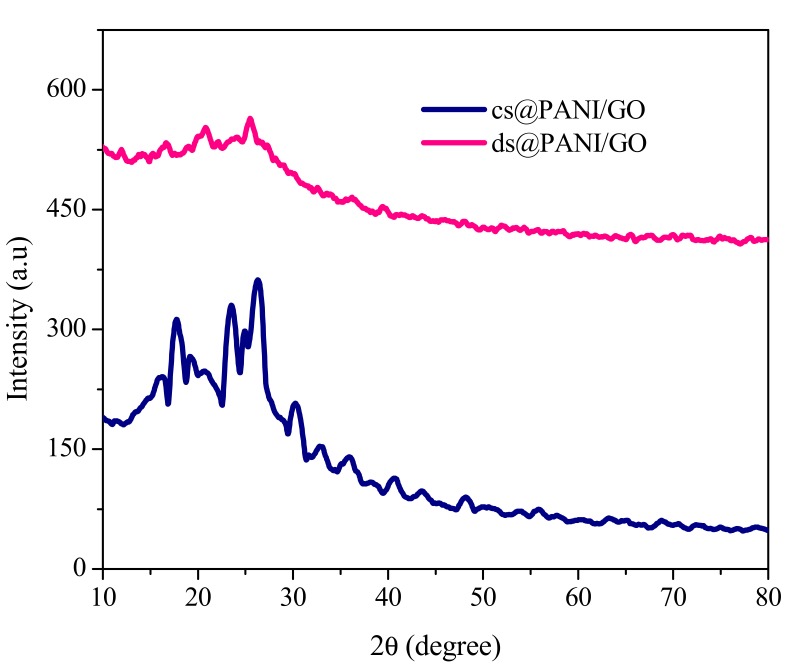
XRD patterns of ds@PANI/GO and cs@PANI/GO.

The XRD patterns of cs@PANI/GO and ds@PANI/GO composites are depicted in Figure 3. The Bragg diffraction shoulders at 2θ = ~15.07°, 2θ = ~20°, and 25.2° appear which are typical peaks of PANI salts [18]. These peaks are characteristic of emeraldine salt form of PANI. The peak at 2θ = ~20° might be due to the recurrence parallel to the polymer chain. The peak at 2θ = ~25.2° can be assigned to the recurrence perpendicular to the polymer backbone. The peak at 2θ = ~20° illustrates the characteristic distance among the ring planes of benzene rings in adjoining chains or the nearby inter-chain gap [19]. Broad peak appears at 2θ = 25.18° in these composites [20]. The XRD pattern of the composites contained reflection peaks that were characteristic of pure PANI, thus depicting prosperous polymerization on the surface of GO [21]. Additionally, the nature of the reflection in these composites were broader, depicting poor sheet ordering along the stacking direction, indicating that the samples could have been entirely exfoliated to contain a few layers of GO [15]. For co-doped cs@PANI/GO, the increase in the intensity of the peak at 2θ = ~26.34°, also the presence of a shoulder, might be due to the higher X-ray scattering factors because of the presence of CSA and GO in this composites [22].

It can be seen from Figure 3, that full-width at half-maximum of the main peaks positioned at 2θ = ~26.34°, 23.29°, and 17.68° for cs@PANI/GO were broader than the corresponding peaks in ds@PANI/GO at 2θ = 25.18°, 16.50°, and 20.72° showing that co-doped ds@PANI/GO had more ordered structure when compared with cs@PANI/GO [18]. It can be assumed that in ds@PANI/GO, PANI chains were aligned in a better way when compared with cs@PANI/GO and possessed enhanced crystallinity.

### 3.4. Morphological Study

**Figure 4:**
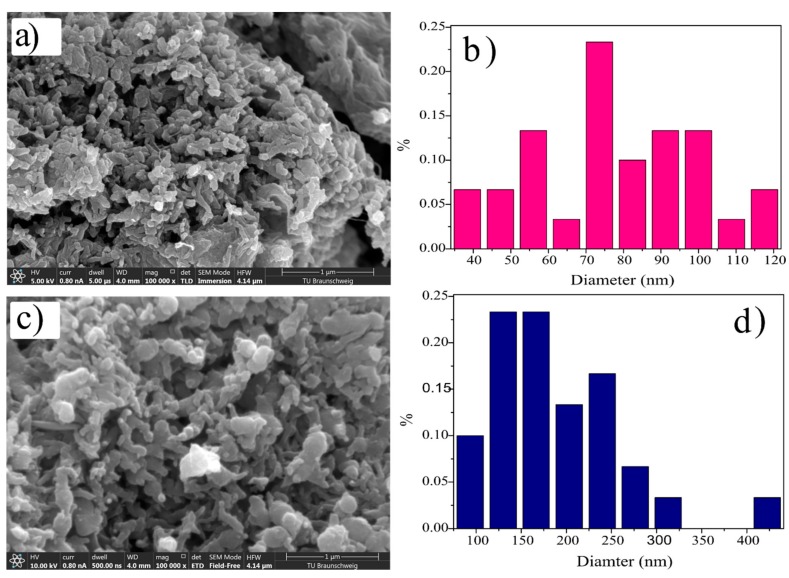
(**a**) SEM image and (**b**) particle size distribution histogram of ds@PANI/GO; (**c**) SEM image and (**d**) particle size distribution histogram of cs@PANI/GO.

The SEM images of the synthesized composites are given in Figure 4a,c. It can be observed from the SEM images that these composites had very small particle size and porous structure. Particle size calculation was done by taking thirty selected particles from each image and plotting diameter versus frequency (Figure 4b,d) and their particle size distribution is given in Table 1.

It can be noticed that the ds@PANI/GO composite had a smaller size compared to the cs@PANI/GO composite, which can be regarded as the influence of the DBSA and GO on morphology [23,24]. Due to the small particle size and porous structure, the overall surface area increased which can ultimately result in an increase in the electrochemical active sites of the electrode material. The contact areas among the electrolyte solution and the active material increases, which is beneficial for the development of the double layer capacitance of graphene oxide components.

### 3.5. Energy Dispersive X-ray Analyis

**Figure 5:**
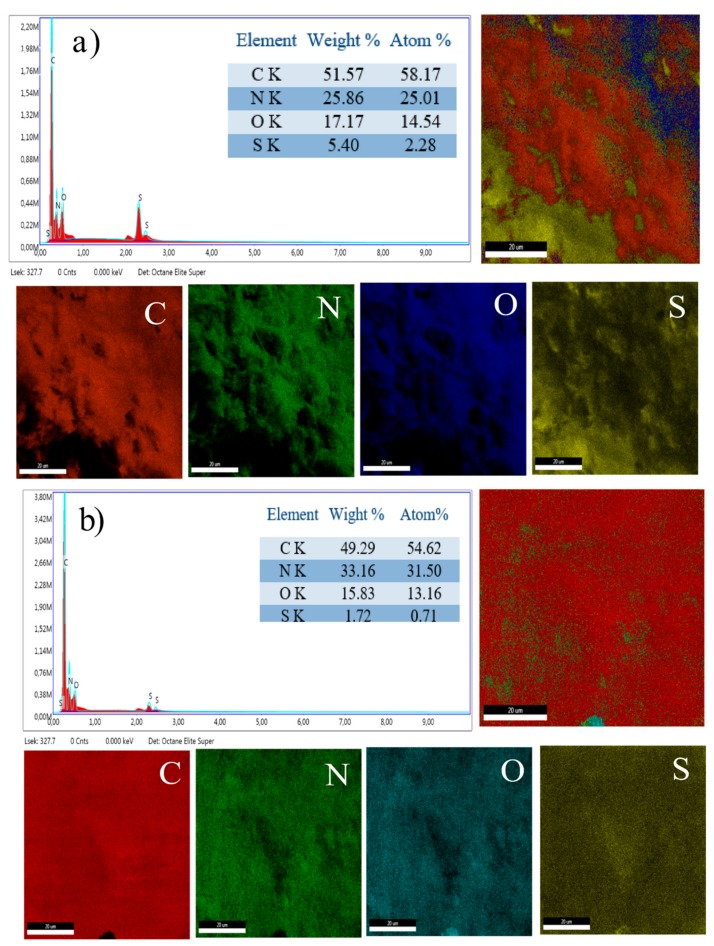
Elemental analysis and mapping of (**a**) ds@PANI/GO and (**b**) cs@PANI/GO.

In order to find different elements and their distribution in the synthesized samples, energy dispersive X-ray (EDX) analysis was carried out and the results are presented in Figure 5a,b. The main components of these composites are C, N, O, and S. Presence of high amount of S in these composites suggest effective incorporation of the dopants. All elements were evenly distributed throughtout the composites. It can be observed in Figure 5a, that in ds@PANI/GO, the S content was higher than in cs@PANI/GO (Figure 5b, indicating a higher level of dopping in ds@PANI/GO. The DBSA molecules in PANI–GO composites were expected to enhance the electrochemical properties of the PANI due to the interaction between the PANI backbone and DBSA alkyl chain, while H_2_SO_4_ will contribute to increase the conductivty of composite materials [25]. These contributions will ultimately lead to low internal resistance of electrode material and, hence, enhanced supercapacitive properties.

### 3.6. Thermogravimetry Analysis (TGA)

**Figure 6:**
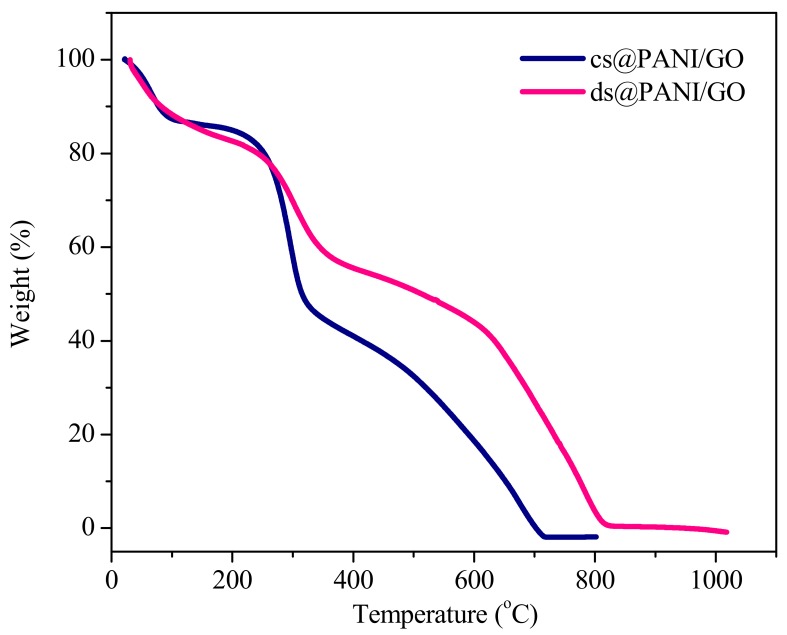
Thermogravimetric analysis (TGA) plots of ds@PANI/GO and cs@PANI/GO.

In Figure 6, TGA plots for ds@PANI/GO and cs@PANI/GO composites are given. Both composites possess typical weight loss steps of PANI.

The first step weight loss was because of the elimination of moisture, while the second weight loss step was because of the elimination of dopant [26]. Graphene oxide containing materials are reported to show a rapid mass loss around 300 °C due to the decomposition of oxygen containing groups, such as –OH, –CO–, and –COOH groups [27].

As can be seen from plot, thermal stability for ds@PANI/GO was shifted to higher temperatures when compared to cs@PANI/GO. This enhanced thermal stability might be due to the better interaction among PANI and GO in ds@PANI/GO due to the presence of DBSA, which facilitates covalent bonding that results in a substantial π–π stacking force among the basal plane of GO and the PANI backbone [28,29].

### 3.7. Electrochemical Characteristics

Electrochemical properties of the synthesized samples were examined with the help of three different techniques including cyclic voltammetry (CV), galvanostatic charge discharge (GCD), and electrochemical impedance spectroscopy (EIS). The electrochemical characteristics of the materials were first tested in three electrode assembly, and then the optimized sample was tested for its performance in symmetric supercapacitor.

#### 3.7.1. Cyclic Voltammetry (CV)

**Figure 7:**
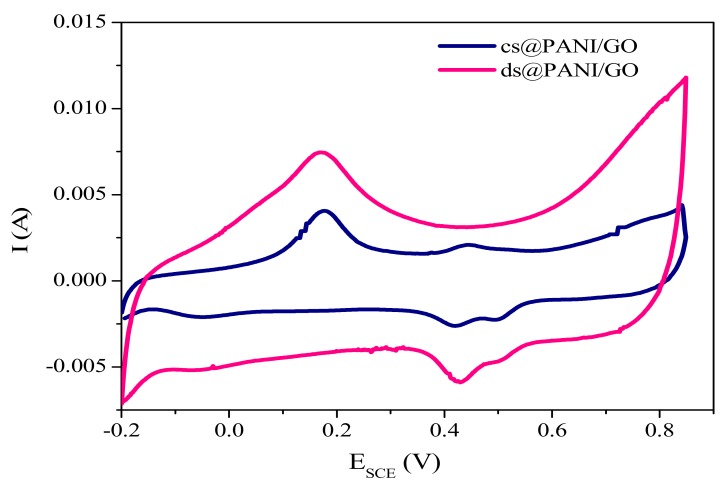
Cyclic voltammogram of ds@PANI/GO and cs@PANI/GO.

Electrochemical assessment of samples was accomplished in a three-electrode setup where 1 M H_2_SO_4_ was utilized as aqueous electrolyte. Figure 7 show CV analysis of ds@PANI/GO and cs@PANI/GO in potential range of −0.2 to 0.85 V for PANI/GO composites. Typical redox peaks of leucoemeraldine/emeraldine and emeraldine/pernigraniline transformation of PANI are present, while a broad capacitive region can also be observed [30].

The area covered by ds@PANI/GO is larger than cs@PANI/GO, once again affirming that presence of DBSA can facilitate better polymerization and improved interaction among different functional groups of GO and PANI. Cyclic voltammetry curves exhibit a rectangular shape overlying a pair of redox peaks, suggesting that the material possess electrical double-layer capacitance (EDLC) and pseudocapacitance simultaneously.

The enhanced peaks in the CV curve of ds@PANI/GO composites arise due to the easy transport of ions onto the surface of the electrode through –SO_3_H groups of DBSA in PANI [31]. These peaks also confirm that the faradic mechanism is also involved. Due to the variations in PANI frameworks and sulfonic acid groups along with oxygenate groups which are attached to GO surface these peaks are more enhanced in ds@PANI/GO. 

#### 3.7.2. Electrochemical Impedance Spectroscopy (EIS)

To gain detailed knowledge about the capacitive properties of composite material and to explore the internal resistance, EIS analysis was carried out. To analyze the performance of composite in the bulk and at interface of electrode and electrolyte, it is a very useful technique to be used. It gives knowledge about the capacitive phenomena that occurs at the electrodes [32].

**Figure 8:**
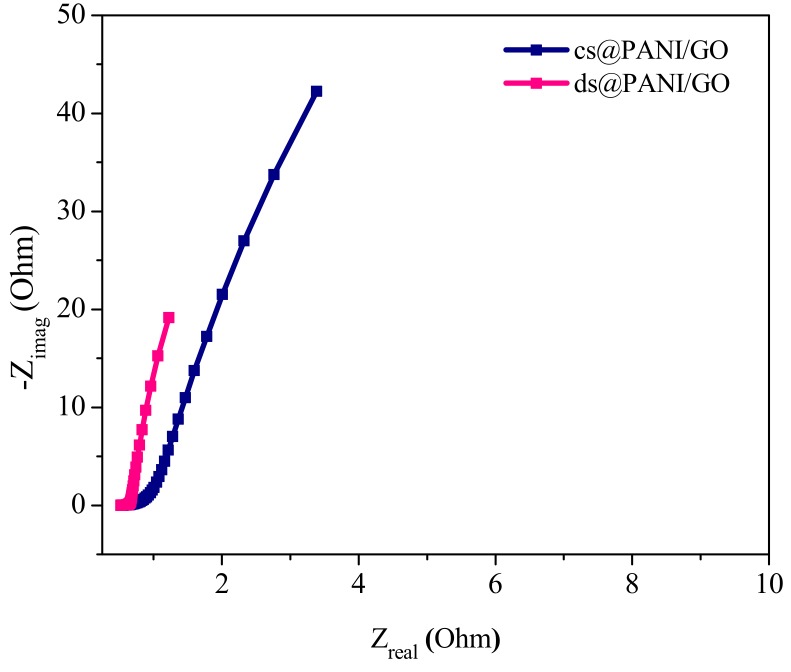
Nyquist plots of ds@PANI/GO and cs@PANI/GO.

The impedance test was performed in the frequency range of 0.05 kHz to 100,000 Hz at the open-circuit potential (Figure 8). The Nyquist plot includes a depressed semicircle in a high-frequency region and a near vertical line in the low-frequency region. The small semicircle shows the good electrical conductivity and low resistance of the material [33,34].

Equivalent series resistance (ESR) for capacitors can be determined from the Nyquist plot where the semicircle on real axis cuts at the high-frequency end [35]. Where Rct, the charge-transfer resistance, can be find out from the radius of the high-frequency arc on the real axis.

It is clear from the plot that both the composites possess low ESR, Rs (solution resistance), and Rct. This might be due to the presence of GO in these composites. The framework provided by GO help the composites sustain the mechanical strength [36]. Moreover, GO can shorten the ion diffusion pathway and create new electroactive regions leading to faster conduction. Additionally, electrical conductivity is also enhanced, resulting in low Rct, which is beneficial for quick charge transfer and facilitate enhanced charge storage.

In low-frequency region vertical arm of the AC impedance illustrates an outstanding capacitive characteristic, which represents very quick ion diffusion and adsorption in/on the electrode material [37]. The impedance curve is about parallel to the imaginary axis in the lower frequency region, illustrating capacitance characteristic. The diversion in slope may be due to the pseudocapacitance of PANI fibers.

It is also noticeable that the arm of the AC impedance in the Nyquist plots of ds@PANI/GO was more vertical than that of cs@PANI/GO, while the values of Rs (0.521 Ohm), ESR (0.661 Ohm), and Rct (0.140 Ohm) were also lower than those of cs@PANI/GO (Rs (0.554 Ohm), ESR (0.927 Ohm) Rct (0.373 Ohm)) [38].

Based on above results, ds@PANI/GO was selected for solid-state symmetric device fabrication due to the fact of its overall good performance. Two types of solid-state symmetric devices were fabricated by utilizing PVA-H_2_SO_4_ Gel as electrolyte and gold or copper as current collectors.

### 3.8. Solid State Symmetric Device Fabrication with Gold as Current Collector

#### 3.8.1. Cyclic Voltammetry

Aqueous and organic electrolytes have been extensively employed for Ucs applications [39]. However, as a consequence of the low-potential window of aqueous electrolyte-based Uc, the energy density is lower than organic electrolyte-based Uc. Flammability, toxicity, and hazardous nature are also the major shortcomings. Additionally, liquid electrolyte-based Ucs needs high-standard safety. Once they leak, it harms our environment. Components part of its configuration are not integrated, which diminishes the electrochemical performance under device movement. Solid-state Ucs have definite benefits when compared with liquid electrolyte-based Ucs, for instance lightweight, good flexibility, high safety and environmental stability, which are crucial for portable devices. The issues, as mentioned above, can be partly eluded by utilizing gel polymer electrolyte as an alternative for aqueous and organic electrolytes. Here, solid-state Uc devices were fabricated using polyvinyl alcohol–H_2_SO_4_ (PVA–H_2_SO_4_) gel electrolyte.

**Figure 9:**
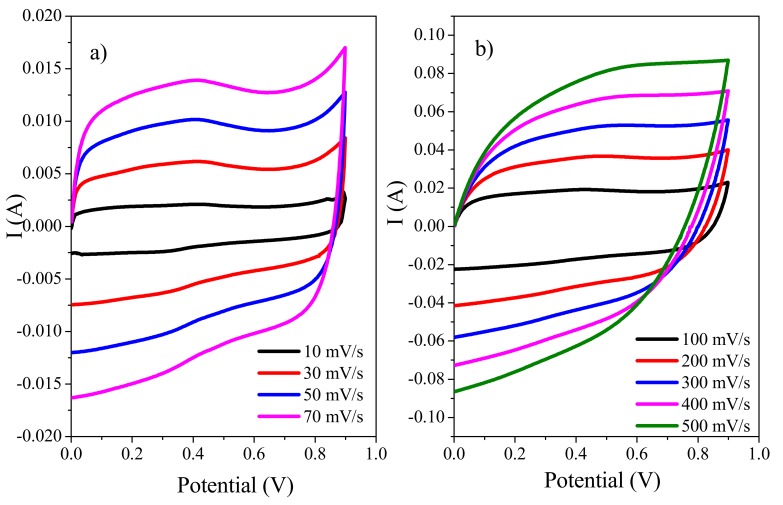
(**a**) CVs of a solid-state symmetric device (using gold as current collector) at various lower scan rates (10, 30, 50, and 70 mV/s) and (**b**) higher scan rates (100, 200, 300, 400, and 500 mV/s).

The CVs of a solid-state symmetric device were recorded in the potential ranges of 0 to 0.9 V at various potential scans (10, 30, 50, 70, 100, 200, 300, 400, and 500 mV/s) as shown in Figure 9a,b. The CVs were almost rectangular shaped with no peaks and maintained the its shape even at high scan rates which indicates the high-rate electrochemical performance [2,40].

#### 3.8.2. Galvanostatic Charge Discharge Analysis (GCD)

**Figure 10:**
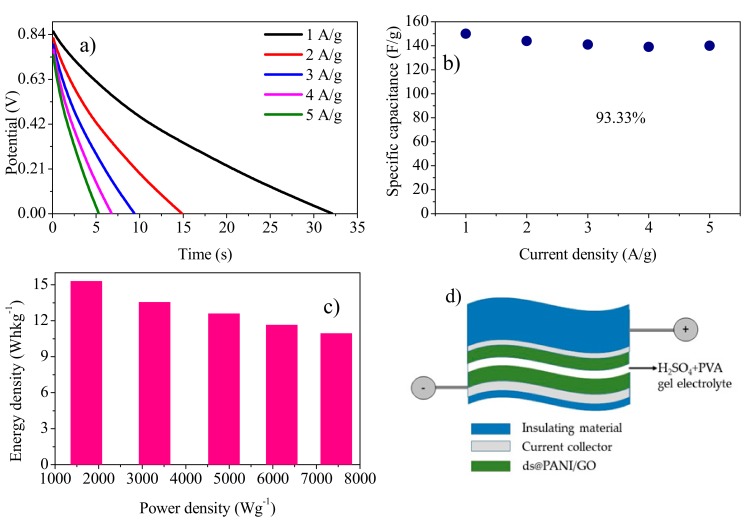
(**a**) Discharge curve of the solid-state symmetric device at various current densities. (**b**) Specific capacitance as a function of current density. (**c**) The Ragone plot. (**d**) Schematic representation of the solid-state symmetric device.

The GCD analysis of the symmetric solid-state device was carried out in the potential range of 0 to 0.9 V at various current densities (Figure 10a). The highest specific capacitance of 150 Fg^−1^ was gained at 1 A^−1^ current density. The value of the capacitance declined with an increase in current density and its value was 140 Fg^−1^ at a current density of 5 Ag^−1^. The value of the capacitance retained at higher current density (5 Ag^−1^) was 93.33% indicating a higher rate capability of the material (Figure 10b) [41] It had a maximum energy density of 15.30 Whkg^−1^ with power density (1716 Wkg^−1^) at 1 Ag^−1^. The schematic representation of the solid-state symmetric device is given in Figure 10d.

**Figure 11:**
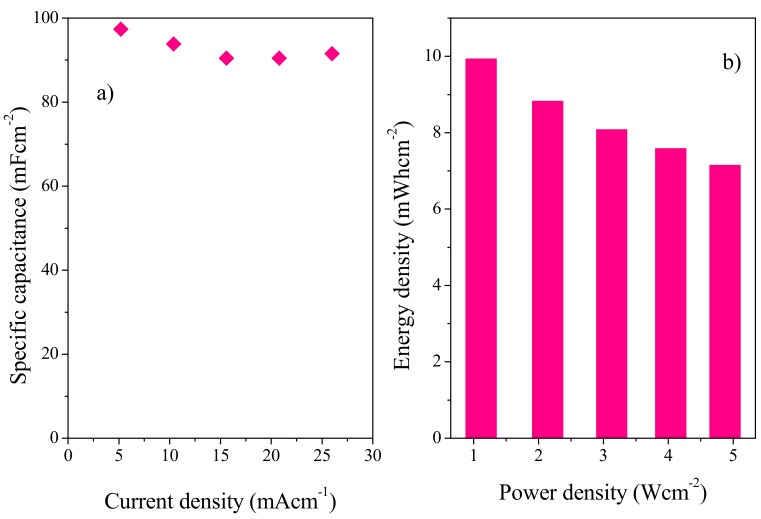
(**a**) Dependence of specific capacitance of the solid-state symmetric device, fabricated with gold as current collector, on current density. (**b**) The Ragone plot.

Areal capacitance was calculated using Equation (5) (Figure 11a). A maximum areal capacitance of 97.38 Fcm^−2^ was achieved at 5 mAcm^−2^, with a power density of 9.93 mWhcm^−2^ and energy density of 1.1 Wcm^−2^ (Figure 11b). These values are quite encouraging as can be seen in Table 2, which summarizes the comparison of the present work with previous studies carried out by other studies.

#### 3.8.3. Electrochemical Impedance Spectroscopy (EIS)

**Figure 12:**
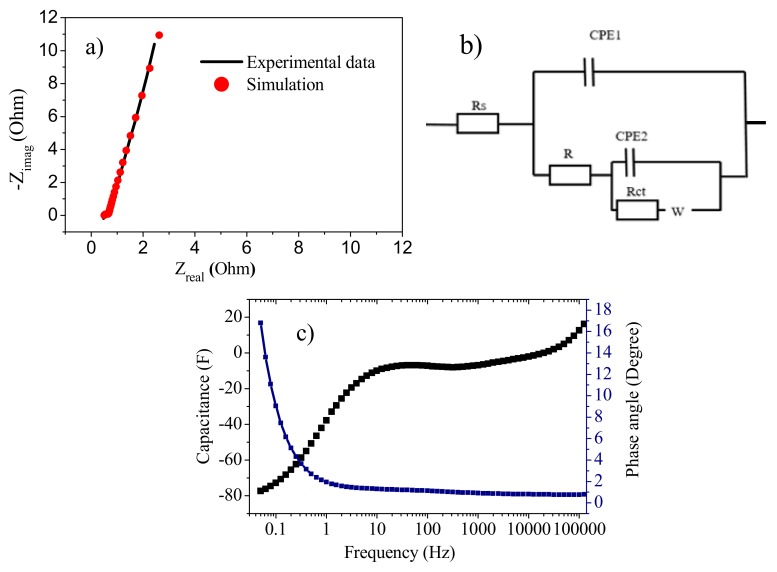
(**a**) Nyquist plot. (**b**): Equivalent circuit model. (**c**) Bode plot of the solid-state symmetric device fabricated with gold as current collector.

To compliment the results from the CV and GCD analysis, EIS was carried out. Figure 12a shows the Nyquist plot with the fitted model. The results of the elements of the equivalent circuit (Figure 12b) are Rs (0.505 Ω), R (0.199 Ω), Rct (0.221 Ω), CPE1 (0.214 S.s^n^), n1 (0.902), CPE2 (0.036 S.s^n^), n2 (0.800), and W (1 × 10^−6^ S.s^1/2^). There was a very small value of W because of the smallest diffusion resistance of the composites [35]. The values of n1 and n2 were much closer to 1 and, thus, their behavior was very near to the ideal capacitor [44].

The Bode plot (Figure 12c) has a phase angle of −77.39° closer to ideal capacitive behavior [45]. Where the t_0_ value at phase angle −45° is 1.42 s. The smaller value of t_0_ means a higher knee frequency and a higher rate capability [46].

### 3.9. Solid State Symmetric Device Fabrication with Copper as Current Collector

#### 3.9.1. Cyclic Voltammetry (CV)

**Figure 13:**
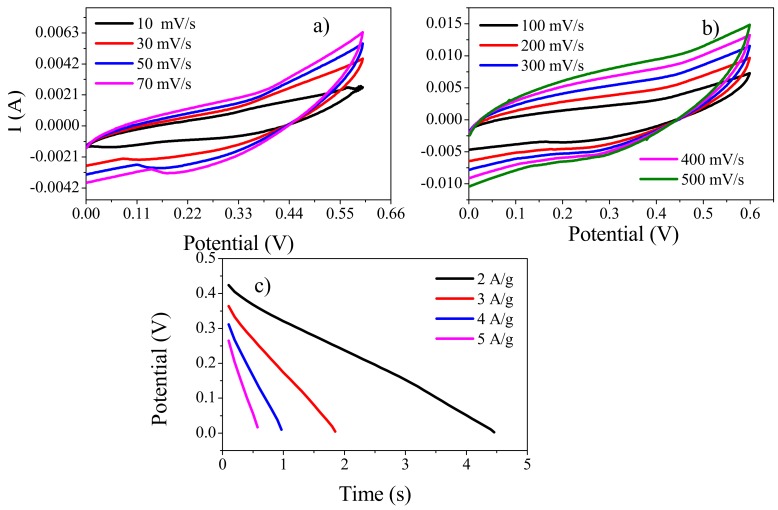
(**a**) CVs of a solid-state symmetric device (fabricated with copper as current collector) at lower scan rates (10, 30, 50, and 70 mV/s) and (**b**) higher scan rates (100, 200, 300, 400, and 500 mV/s). (**c**) Discharge curve of the same device at different current densities using copper as current collector.

Due to the many advantages of solid-state Ucs, copper sheets were also used as current collectors to fabricate solid-state symmetric device, as it is much cheaper than gold. Cyclic voltammetry analysis was accomplished in the potential range of 0 to 0.6 V at various scan rates of 10, 30, 50, 70, 100, 200, 300, 400, and 500 mV/s (Figure 13a,b). These CVs maintained their shape even at high scan rate, thus, showing high rate capability. These results depict that the synthesized material can also be used as Uc electrode material when cheaper material such as copper is used as current collector and, so, the cost of solid-state Ucs can be reduced [47].

#### 3.9.2. Galvanostatic Charge Discharge (GCD) Analysis

The GCD analysis of the device, fabricated with copper as current collector, was executed in the potential range of 0 to 0.6 V at several current densities. As can be seen from Figure 13c, with the decrease in current density, the discharge time increased. Also, the values of IR drop, raising with an increase in current density. The specific capacitance values were 82, 58, 46, and 38 F/g at 2, 3, 4, and 5 A/g current densities, respectively. The maximum value (82 F/g) was obtained at 2 A/g that decreased to 38 F/g at 5 A/g current density. These values were, however, lower than those obtained when gold was used as current collectors. The results also suggest that the current collector does play a role in the overall performance of the device; therefore, we plan to look for current collectors that are not only economical but can also sustain good performance of the device.

## 4. Conclusions

Solid-state symmetric Uc devices were fabricated by utilizing gold or copper as current collectors, PAV-H_2_SO_4_ gel as electrolyte, and two different PANI–GO composites as electrode materials. There was difference in the overall performance of the device when copper was used as current collector instead of gold, indicating that the current collectors do contribute to the performance of the device. The use of PAV–H_2_SO_4_ gel electrolyte makes the device eco-friendlier by minimizing the risk of electrolyte leakage. While the difference in dopants also showed a pronounced effect on the properties of the composite generally and its overall performance as electrode material in the device specifically. Contributions from pseudo capacitance of PANI and EDL capacitance of GO were visible in the composite material while DBSA or CSA as dopants in combination with H_2_SO_4_ also affected the results. Apparently all the combined effects contributed to the enhanced capacitance, energy density, and power density of the fabricated device. Best performance was achieved the for ds@PANI/GO composite where DBSA and H_2_SO_4_ were used as dopants. Highest specific capacitance value of 150 F/g was achieved at 1 A/g current density. The device showed excellent rate capability with capacitance retention of 93.33% at higher current density (10 A/g). At 1 A/g, maximum energy density of 15.30 Whkg^−1^ with a power density of 1716 Wkg^−1^ was obtained. Its maximum areal capacitance was 97.38 mFcm^−2^ at 5 mAcm^−2^. The device fabricated with copper as current collector, also manifested capacitive response and showed 82 F/g specific capacitance value at 2 A/g current density. However, its performance was lower than the device fabricated with gold as current collector.

## Figures and Tables

**Table 1:** Particle size distribution of ds@PANI/GO composite and cs@PANI/GO composite.

S. No	Sample Name	Total	Max./nm	Min./nm	Mean/nm
1	ds@PANI/GO composite	30	121.53	34.22	78.74
2	cs@PANI/GO composite	30	440.55	75.19	190.05

**Table 2:** Comparison of the performances of the fabricated device with similar previous works.

S. No	Electrode Material	Electrolyte	Potential Window (V)	Current Density (A/g)	Specific Capacitance	Reference
1	Symmetrical PANI/Au/PEN	PVA/H_2_SO_4_ gel	0–0.8	0.15 mA cm^−2^	29 mF cm^−2^	[42]
2	PANI/rGO–TA–24 h	H_2_SO_4_–PVA	0–0.5	0.5 A g^−1^	56.9 F g^−1^	[43]
3	PANI/rGO–HH	H_2_SO_4_–PVA	0–0.5	0.5 A g^−1^	46.0 F g^−1^	[43]
4	Co-doped ds@PANI/GO	PVA/H_2_SO_4_ gel	0–0.9	1 Ag^−1^	150 Fg^−1^	Present work
5	Co-doped ds@PANI/GO	PVA/H_2_SO_4_ gel	0–0.9	5 mAcm^−2^	97.38 Fcm^−2^	Present work
